# Nocturnal finger skin temperature in menstrual cycle tracking: ambulatory pilot study using a wearable Oura ring

**DOI:** 10.1186/s12905-019-0844-9

**Published:** 2019-11-29

**Authors:** Anna Maijala, Hannu Kinnunen, Heli Koskimäki, Timo Jämsä, Maarit Kangas

**Affiliations:** 1 Research Unit of Medical Imaging, Physics and Technology, University of Oulu, Oulu, Finland; 2 Oura Health, Oulu, Finland; 3 Optoelectronics and Measurement Techniques Research Group, University of Oulu, Oulu, Finland; 4 Biomimetics and Intelligent Systems Group, University of Oulu, Oulu, Finland; 5 Medical Research Center, University of Oulu and Oulu University Hospital, Oulu, Finland; 6 Department of Diagnostic Radiology, Oulu University Hospital, Oulu, Finland

**Keywords:** Ovulation, Menstruation, Follicular phase, Luteal phase, Oral temperature, women’s health

## Abstract

**Background:**

Body temperature is a common method in menstrual cycle phase tracking because of its biphasic form. In ambulatory studies, different skin temperatures have proven to follow a similar pattern. The aim of this pilot study was to assess the applicability of nocturnal finger skin temperature based on a wearable Oura ring to monitor menstrual cycle and predict menstruations and ovulations in real life.

**Methods:**

Volunteer women (*n* = 22) wore the Oura ring, measured ovulation through urine tests, and kept diaries on menstruations at an average of 114.7 days (SD 20.6), of which oral temperature was measured immediately after wake-up at an average of 1.9 cycles (SD 1.2). Skin and oral temperatures were compared by assessing daily values using repeated measures correlation and phase mean values and differences between phases using dependent t-test. Developed algorithms using skin temperature were tested to predict the start of menstruation and ovulation. The performance of algorithms was assessed with sensitivity and positive predictive values (true positive defined with different windows around the reported day).

**Results:**

Nocturnal skin temperatures and oral temperatures differed between follicular and luteal phases with higher temperatures in the luteal phase, with a difference of 0.30 °C (SD 0.12) for skin and 0.23 °C (SD 0.09) for oral temperature (*p* < 0.001). Correlation between skin and oral temperatures was found using daily temperatures (r = 0.563, p < 0.001) and differences between phases (r = 0.589, *p* = 0.004). Menstruations were detected with a sensitivity of 71.9–86.5% in window lengths of ±2 to ±4 days. Ovulations were detected with the best-performing algorithm with a sensitivity of 83.3% in fertile window from − 3 to + 2 days around the verified ovulation. Positive predictive values had similar percentages to those of sensitivities. The mean offset for estimations were 0.4 days (SD 1.8) for menstruations and 0.6 days (SD 1.5) for ovulations with the best-performing algorithm.

**Conclusions:**

Nocturnal skin temperature based on wearable ring showed potential for menstrual cycle monitoring in real life conditions.

## Background

An important use case for menstrual cycle monitoring is fertile window tracking, whether the aim is to get pregnant or to avoid pregnancy. Besides fertility awareness, women’s motivations to monitor menstrual cycle include various other reasons, such as being prepared for an upcoming menstruation or understanding different body reactions in different cycle phases [1]. Menstrual cycle also affects other areas of women’s lives, such as sleep [2], sensitivity to drugs [3], craving for smoking [4], craving for food and food intake [5], and subjective stress responses [6], and causes symptoms such as irritability, depressed mood, swelling, and headache in premenstrual phase.

Fertility awareness-based methods including both non-usage of technology, such as different calendar methods and following vaginal secretion, and usage of technology, such as basal body temperature (BBT) measurements, are based on the following of physical symptoms experienced throughout the menstrual cycle [7]. Women’s BBT curve is typically biphasic, as the temperature is about 0.2–0.4 °C higher in the luteal (post-ovulation) phase compared to the follicular (pre-ovulation) phase. Progesterone, as a thermogenic hormone, is considered to be responsible for the rise in BBT that typically occurs at 1–3 days post ovulation. Though utilizing body temperature is mostly used in detection of fertility related phases, few studies have also used it in prediction of menstrual cycle length or the start of menstruation [8, 9].

Rectal temperature is considered to represent core body temperature (CBT). CBT has a circadian rhythm, that is, a daily fluctuation in which the lowest temperature during rest is considered to represent BBT. Though it has been summarized that oral temperature cannot be used to reflect CBT [10], oral body temperature measured immediately after wake-up has been proven to vary during menstrual cycle with higher temperatures in the luteal phase [11] and is widely used as a practical alternative to measure BBT in home environments among clinically tested fertility tracking applications and devices such as cycle computers [12–14].

The circadian rhythm of distal skin temperature has also been studied under both constant routine protocols and ambulatory study conditions. Distal skin temperature has been reported to have wide maximums, whereas CBT has its wide minimums during the night-time [15], CBT peak sometimes lagging for 180 min [16]. The circadian rhythm of wrist skin temperature has similarly shown the highest values during sleep and the lowest when awake [17–19]. Already laying down causes increase in distal skin temperature [17].

Most of the studies using different kinds of skin-attached temperature sensors assessing menstrual cycle and conducted under laboratory conditions with a few hours of measurement time once or twice per follicular and luteal phase have not found any differences in skin temperatures between menstrual cycle phases [20, 21]. However, in ambulatory studies, skin temperatures have been found to have a biphasic temperature property, with higher values in the luteal phase and lower values in the follicular phase [9, 11], and to be in phase with oral temperature measured in the morning [11].

Nowadays wearable sensors such as armbands, bracelets, and earbuds measuring nocturnal temperature have entered fertility awareness-based methods and industry offering more effortless ways to track menstrual cycle. To date, only a limited amount of studies have been conducted using these wearables. A wrist-worn armband detected biphasic skin temperature patterns in 82% of the ambulatory measured menstrual cycles with significantly higher average temperatures in early-luteal and late-luteal phases compared to the menstrual phase and 86% of the BBT shifts occurring after ovulation [22]. The most recent studies utilizing wearables in ovulation or fertile window detection and prediction have utilized also different kind of statistical models [23] and machine learning algorithms [24]. However, performance comparison to accurate reference measurements is lacking [25].

To our knowledge, no earlier ambulatory studies using finger skin temperature in menstrual cycle phase tracking have been conducted. The first aim of this pilot study was to assess the applicability of nocturnal finger skin temperature measured by the wearable Oura ring to monitor menstrual cycles in real life. This was done by comparing skin temperature between menstrual cycle phases and correlating skin temperature with oral temperature measured immediately after wake-up. Part of the results have been published earlier [26]. The second aim was to develop and evaluate algorithms utilizing skin temperature for predicting the start of menstruation and ovulation.

## Methods

### Participants

Volunteer women (*n* = 31) were recruited using information channels of different work and hobby communities to participate in a larger study including physical activity, menstrual cycle, and subjective feelings of readiness and sleep. Inclusion criteria were (1) female, (2) minimum age of 18 years, and (3) mobile phone’s operating system supported by Oura application. Exclusion criteria were (1) bypassed menopause, or (2) pregnant in the beginning of the study. This was a sub-study with additional exclusion criteria (3) hormonal contraception (*n* = 8). In addition, one test subject using progesterone medication was excluded resulting in a total number of 22 test subjects for this study (Table 1). The enrolled women did not get any payment to enter the study but those who completed the measurements were offered the possibility to continue the use of the Oura ring after the study. As a commercial product the Oura ring gave the participants health-related information such as summary of their sleep and physical activity.

**Table 1:** Characteristics for participants (*n* = 22)

Characteristic	
Age (years), average (SD), range	34.7 (8.8), 21–49
BMI (kg/m^2^), average (SD), range	24.3 (3.6), 20.3–37.2^a^
Contraceptive method, n (%)	
none	11 (50.0)
non-hormonal	11 (50.0)
not mentioned /	7 (31.8)
condom /	3 (13.6)
copper intra uterine device	1 (4.5)
Regular menstruation, n (%)	19 (86.4)
Underlying diseases, n (%)	4 (18.2)^bb^
Continuous medications, n (%)	4 (18.2)^ccc^
Smoking, n (%)	0 (0.0)

^a^BMI > 30: two participants

^bb^none were affecting temperature nor menstrual cycle

^ccc^depression medication (one participant); others did not have medications with potential effect on temperature or cycle

The study participants were provided written and oral information about the procedures of the study, and written consent was obtained. The study was conducted in accordance with the Declaration of Helsinki. An ethical statement (2/2017) was obtained from the Ethics Committee of Human Sciences, University of Oulu, Finland. Subjects could suspend their participation in the study at any point.

Out of 22 participants, 3 dropped out before the end of the study. However, the consent enabled the use of data until drop-out, and their data until drop-out were included in the analyses. In addition, one participant started using hormonal contraceptives, and only data measured before this was analyzed. Four participants had considerable amount of missing daily skin temperature values (over 50%) during some menstrual cycles, and thus only the longest continuous part of their data was included in the analyses.

### Study protocol, measurements, and parameters

The study protocol was designed to collect data from 120 to 150 days from each participant to obtain data from three full menstrual cycles. The study took place between late spring and early autumn in 2017 in Finland.

In the first day of the study, participants answered a preliminary survey with questions related to menstruation as well as their general background info. All information including weight and height were asked from the participants.

Participants wore the Oura ring, a commercially available wearable sleep and activity tracker (Oura Health, Oulu, Finland), on their freely selected finger around the clock. The ring could also be used in water (up to 50 m deep) and in sauna, but it was instructed not to be left off-finger in hot spaces. Lifting heavy weights while wearing the ring was not recommended. The Oura ring has a negative temperature coefficient (NTC) thermistor (non-calibrated, resolution of 0.07 °C) as its temperature sensor. The sensor has been programmed to register skin temperature readings from the palm side of the finger base every minute when the ring is worn. The ring can be connected to a mobile phone application, Oura, via Bluetooth. In the beginning of the study, each participant downloaded the Oura application from either the Google Play Store or the Apple App Store to their mobile phones and created an Oura account. Participants were able to view their own data provided by the application. Participants were asked to open the application every morning to upload the data from the ring to the application. Uploaded data was automatically transferred via Internet connection to the study database in the Oura cloud service.

A MATLAB script was programmed (MATLAB R2017b) to determine a representative nightly temperature value from the minute-by-minute skin temperatures. The script was developed using skin temperature data measured with the Oura ring in a separate pilot study with 11 women (average age 32.9 years, SD 10.6) (unpublished data). A moving average filter with a length of 17 min was applied to nocturnal temperature data (from 10:00 p.m. to 8:00 a.m.). Filtered values were defined as stable if the fluctuation within values used in its calculation was less than 1 °C. The highest stable filtered temperature was used as nightly temperature. If no stable temperature was found, the daily temperature value was defined missing.

Oral body temperature was measured using a thermometer (Omron Ecotemp Basic, Omron Health Care Inc) immediately after wake-up before getting out of bed for at least one menstrual cycle length. The thermometer gave temperature values digitally with a technical accuracy of ±0.1 °C.

Ovulation days were detected using commercial urine test for luteinizing hormone (LH) (One Step Ovulation Test Midstream (Urine), AI DE Diagnostica Co. Ltd.) with concentration threshold 20 mlU/ml (accuracy of 99% based on manufacturer’s information). Ovulation tests were instructed to be performed every 12 h starting on the menstrual cycle day specified in the ovulation test instructions. The day following the first positive ovulation test result was used as a verified ovulation day (day 0) based on the interpretation guidance of the test instructions and literature [27]. Participants recorded the measured oral body temperature values and ovulation test results on a paper diary. In addition, participants kept diaries of menstruation days and menstrual cycle days throughout the whole study.

#### Applicability evaluation

The applicability of nocturnal finger skin temperature in monitoring menstrual cycle was tested in two ways by comparing oral thermometer and skin measurements (1) over the course of time and (2) between menstrual cycle phases. Oral temperature was measured immediately after wake-up. The menstrual cycle period before ovulation was defined as the follicular phase and after as the luteal phase. The first day of menstrual flow was used as the start of the follicular phase. In both temperature comparisons, only cycles with positive ovulation test results and at least 50% of temperature data for both measurement methods and each phase were analyzed. The temperature data of these cycles were used to calculate participants’ mean and standard deviation (SD) temperatures for each phase and method.

#### Algorithm testing

Preprocessing of daily skin temperature values was done with the following steps using a MATLAB script (MATLAB R2017b).

1. Filling in the missing daily temperature values with linear interpolation.
2. Finding the menstrual cycle component. Based on the literature, the average cycle length is 28 days. This was used in a filter design to differentiate the biphasic menstrual cycle component from the daily temperature data. Filtering was done using 2-order Butterworth low-pass filter with a cutoff frequency of 1.5/28 samples/day and a sampling frequency of 1 sample/day.
3. Finding locations, that is, days, of minimums (MIN) and maximums (MAX) of the menstrual cycle component by starting from the MIN with the lowest temperature or the MAX with the highest temperature, and using a minimum peak distance of 15 days for two minimums or two maximums. The average menstrual cycle length (AVG_MCL) was defined by calculating the average distance between two successive minimums and two successive maximums using all minimums and maximums.

The temperature values of the menstrual cycle component were used by all the algorithms in menstrual cycle phase tracking. The ovulation day is related to the rise in daily temperature and the start of menstruation to the decline in daily temperature [9].

**Fig. 1:**
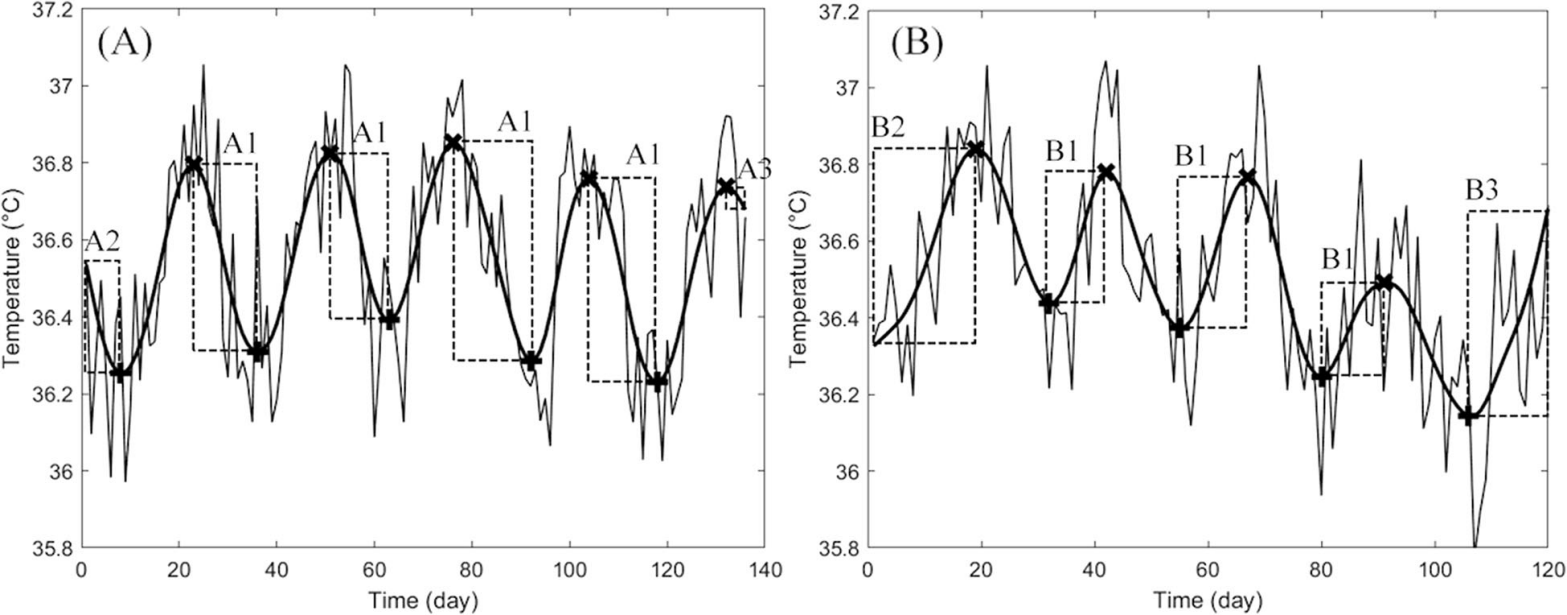
Example skin temperature data with search limits for tracking (**a**) start of menstruation and, (**b**) ovulation. The narrow solid line represents the daily temperature values. The thick solid line represents the fitted menstrual cycle component and marks x and + maximums and minimums of the fitted component, respectively. Search limits are presented as dashed rectangles A1-A3 and B1-B3. The algorithm for tracking the start of menstruation used A1-A3. The algorithms for ovulation tracking used the following search limits: HALF_LOCS, B1-B3; HALF_PEAKS, B1; and RISE_0.15, B1 and B3

Based on our separate pilot study, the start of menstruation was associated with the middle time point between the adjacent MAX and MIN ((MAX + MIN) / 2) of the fitted component. Thus, in this study, the start of menstruation was predicted to (MAX + MIN) / 2 rounded down (algorithm MENSES), that is, the middle day between the adjacent MAX and MIN (search limit A1 in Fig. 1a). For the data analyses of the beginning and the end of the data where the full length of data for MAX and MIN was not available, the estimation of the start of menstruation was based on estimating the middle time point using the average cycle length determined from the data. Time points (MIN - (AVG_MCL / 4) rounded down) and (MAX + (AVG_MCL / 4) rounded down) were used for the beginning (search limit A2 in Fig. 1a) and the end (search limit A3 in Fig. 1a), respectively.

Three algorithms predicting the ovulation day were defined. The algorithm HALF_LOCS predicted ovulation similarly to the algorithm MENSES as the middle day between the adjacent MIN and MAX (search limit B1 in Fig. 1b), that is, (MIN + MAX) / 2 rounded down. For the data analysis of the beginning and the end of data where the full length of data for MIN and MAX was not available, the estimation of ovulation was based on approximating the middle time point using the average cycle length determined from the data. Time points (MAX - (AVG_MCL / 4) rounded down) and (MIN + (AVG_MCL / 4) rounded down) were used for the beginning (search limit B2 in Fig. 1b) and the end (search limit B3 in Fig. 1b), respectively.

At the simplest, ovulation prediction is based on detecting a certain absolute rise in temperature [28]. This was applied to the existing data with two algorithms. The algorithm HALF_PEAKS predicted the ovulation day as the first day between the adjacent MIN and MAX (search limit B1 in Fig. 1b) that had a temperature value greater than the average temperature of these MIN and MAX. The algorithm RISE_0.15 predicted the ovulation day as the first day between the adjacent MIN and MAX (search limit B1 in Fig. 1b) or between MIN and the last data value (search limit B3 in Fig. 1b) that had a temperature value of at least 0.15 °C greater than the MIN. Ovulation days were also predicted using a biological rhythm -based method for practical use. Menstrual cycle length was approximated as the average length of the preceding menstrual cycles during the study. If there was no data on previous cycle lengths, the average cycle length reported by the participant in the preliminary survey was used. Ovulation was estimated to occur 14 days before the predicted last day of the cycle.

### Statistical analyses

For applicability evaluation, the comparison between skin and oral temperatures was assessed using RStudio version 1.1.453 (RStudio, Inc.) for repeated measures correlation (rmcorr) [29] using daily temperature values, and SPSS Statistics version 24 (IBM) for dependent t-test with 95% confidence interval using the mean temperature of the luteal phase (ML) and the mean temperature of the follicular phase (MF), and the difference between ML and MF.

For algorithm testing, sensitivities and positive predictive values (PPV) of predicting menstruations and ovulation days were calculated as Eqs. 1 and 2, respectively. Predicted start of menstruations and ovulation days for the algorithms were defined as true positives (TP) or false positives (FP) using windows ±1, ±2, ±3 and ± 4 days around the reported start of menstruation, and windows ±1, ±2, − 4 to + 1, − 3 to + 2 days around the verified ovulation day for estimation error. The two last-mentioned 6-day-windows were defined on the basis of fertility point of view: depending on the reference ovum living up to 1–2 days after ovulation, and sperm surviving for 6 days inside women’s body. Menstruation or ovulation predicted within the window was considered as TP and outside as FP. Negative estimation error indicated the estimate to precede the reported or verified day and the positive to lag. If ovulation detection with LH test failed or ovulation was not measured, FP was left out of analyses. Reported start of menstruation or verified ovulation that was not detected by the algorithm within the window was defined as FN. Estimations and false negatives with a full length of data required by the algorithm based on its search limits and window were analyzed.

1 $$ Sensitivity=\frac{TP}{TP+ FN}\ast 100\% $$

2 $$ \mathrm{P} PV=\frac{TP}{TP+ FP}\ast 100\% $$

## Results

The total study length was on average 114.7 days (SD 20.6, range 43–150). Participants (*n* = 22) had a total of 99 menstruations and 80 verified ovulations during the study. On average, participants had 4.5 menstruations (SD 0.9) and 3.6 verified ovulations (SD 1.1). Menstrual cycle length varied between 21 and 50 days (mean 27.6, SD 4.4) and the day with the first positive ovulation test result varied between cycle days 9 and 31 (average 13.9, SD 4.3). The total number of menstrual cycles in the temperature comparisons was 41 and 1.9 per participant (SD 1.2) with average daily temperature availability per menstrual cycle for ring measurements being 97.4% (range 83.3–100.0%) and for oral measurements being 92.9% (range 60.6–100.0%). In total, the daily skin temperature availability before filling in the missing values was on average 96.6% per participant (range 80.7–100.0%).

### Applicability evaluation

**Fig. 2:**
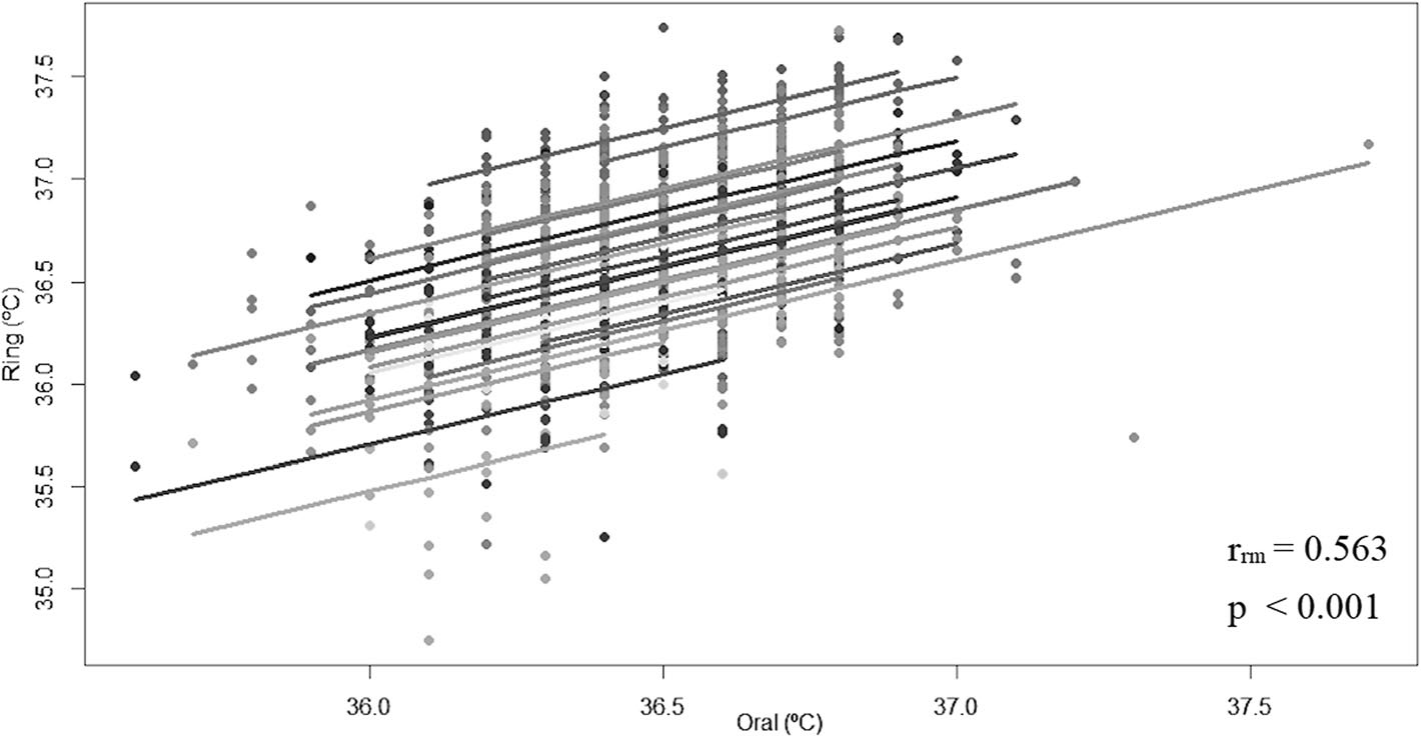
Rmcorr plot of daily temperature values from the oral thermometer and the Oura ring

Nocturnal skin temperature maximums based on the ring and oral temperatures measured immediately after wake-up were correlated by r = 0.563 (*p* < 0.001, degrees of freedom 992, 95% CI 0.519–0.604) (Fig. 2).

**Fig. 3:**
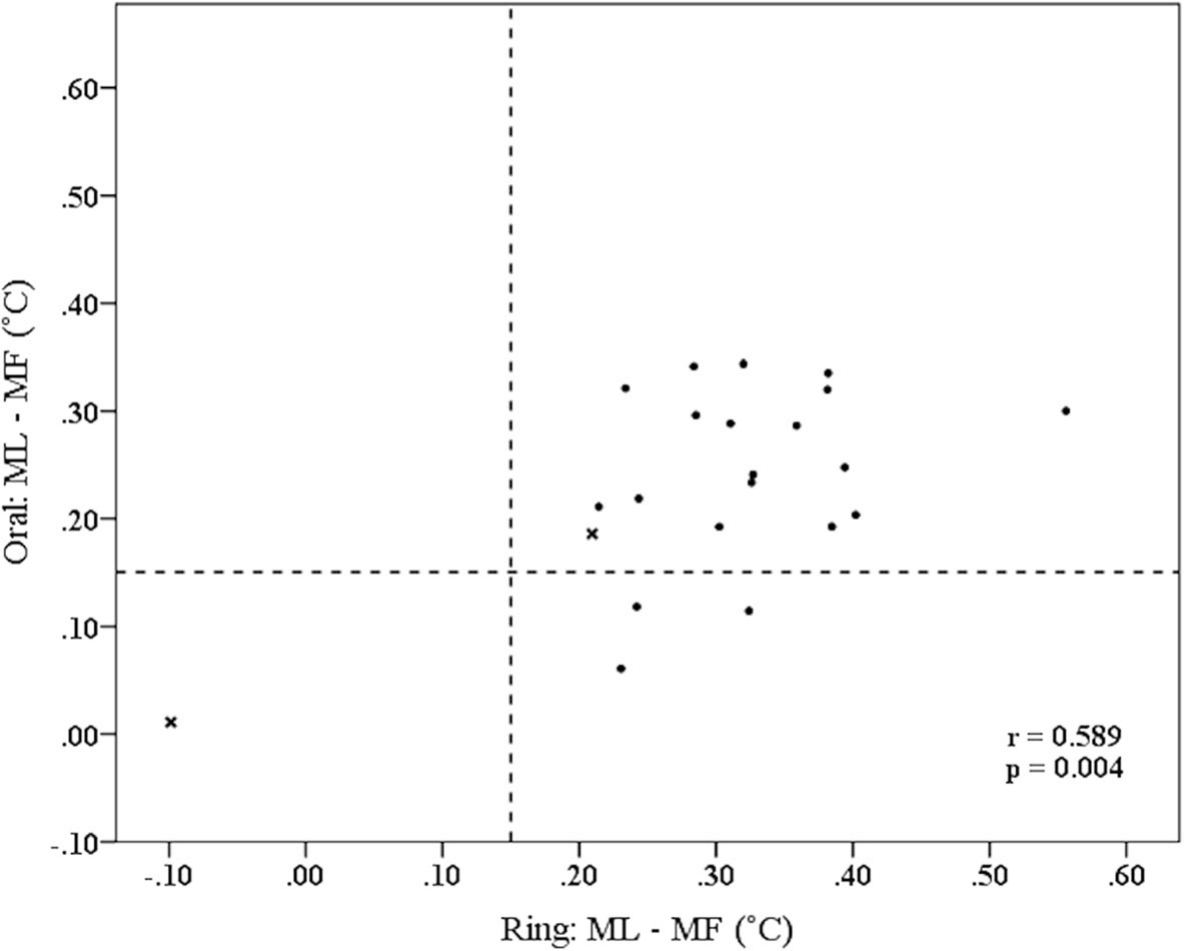
Scatter plot of menstrual phase based mean temperature values from the oral thermometer and the Oura ring. Dashed lines depict 0.15 °C difference between the phases (the criterion used in RISE_0.15). Test subjects with BMI over 30 marked as x

Skin temperature measured with the ring and oral temperature both differed between the luteal phase and the follicular phase (Fig. 3): the difference between the mean of the phases was 0.30 °C (SD 0.12, p < 0.001) and 0.23 °C (SD 0.09, p < 0.001) for skin and oral temperature, respectively. Temperature difference was higher with skin temperatures than with oral temperatures (average 0.07 °C, SD 0.10, *p* = 0.003). In skin temperature measurements, 21/22 participants met the pre-defined requirement of 0.15 °C difference between the phases (1 not meeting had irregular cycles) (Fig. 3). In oral temperature measurements, 18/22 participants met the requirement. Two of those not meeting the requirement had irregular cycles. The difference between ML and MF correlated by r = 0.589 (*p* = 0.004) (Fig. 3).

The average standard deviation of daily temperature values was 0.20 °C (SD 0.07) in the follicular phase and 0.24 °C (SD 0.08) in the luteal phase for skin and 0.17 °C (SD 0.05) in the follicular phase and 0.19 °C (SD 0.07) in the luteal phase for oral temperatures.

### Algorithm testing

**Fig. 4:**
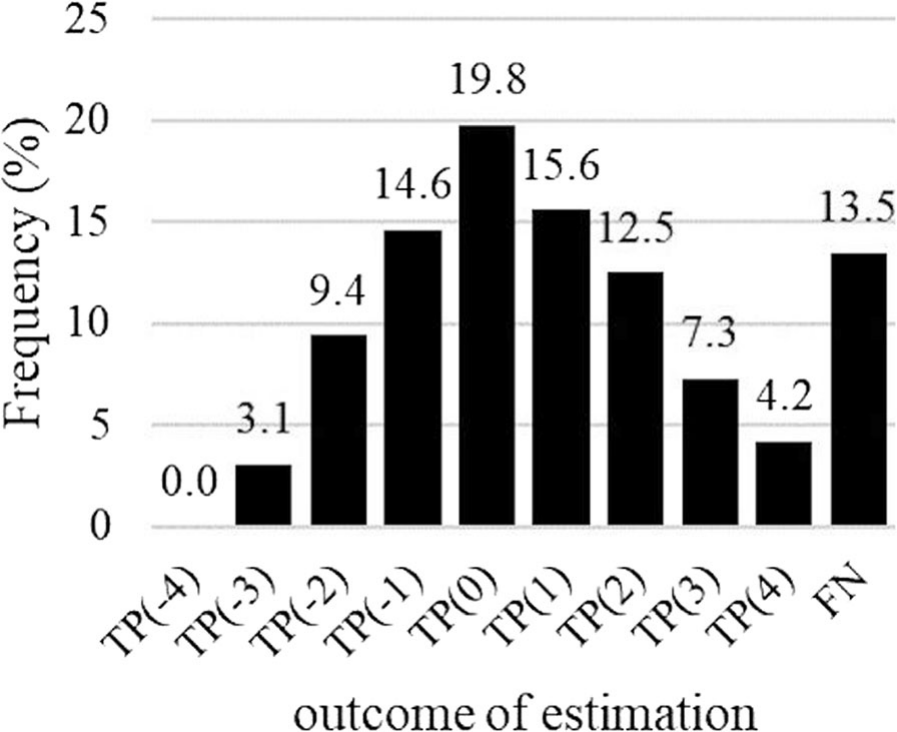
Menstruation prediction using algorithm MENSES. The distribution of detected menstruations (TP) in window ±4 days around the reported day relative to all reported menstruations (TP + FN = 96). FN represents menstruations not detected within the window

The performance of each algorithm was evaluated by testing for sensitivity and PPV using the skin temperature data. Algorithm MENSES detected 19.8% of the start of menstruations on the reported day and 50% within ±1 days (Fig. 4). The mean offset from the reported day for the start of menstruation was 0.4 days (SD 1.8) for the algorithm when the true positives in the window length of ±4 days from the reported day were used. The sensitivity of the algorithm reached 81.4 and 86.5% with window lengths of ±3 and ± 4 days, respectively (Table 2).

**Table 2:** Menstruation prediction: performance of algorithm MENSES

Window (days)	Menstruations (TP + FN)^a^	Sensitivity (%)	PPV (%)
±4	96	86.5	85.6
±3	97	81.4	81.4
±2	96	71.9	71.1
±1	96	50.0	49.5

^a^The differences in the number of TP + FN are caused by different data availability requirements of the different windows. *TP* true positive, *FN* false negative, *PPV* positive predictive value

**Fig. 5:**
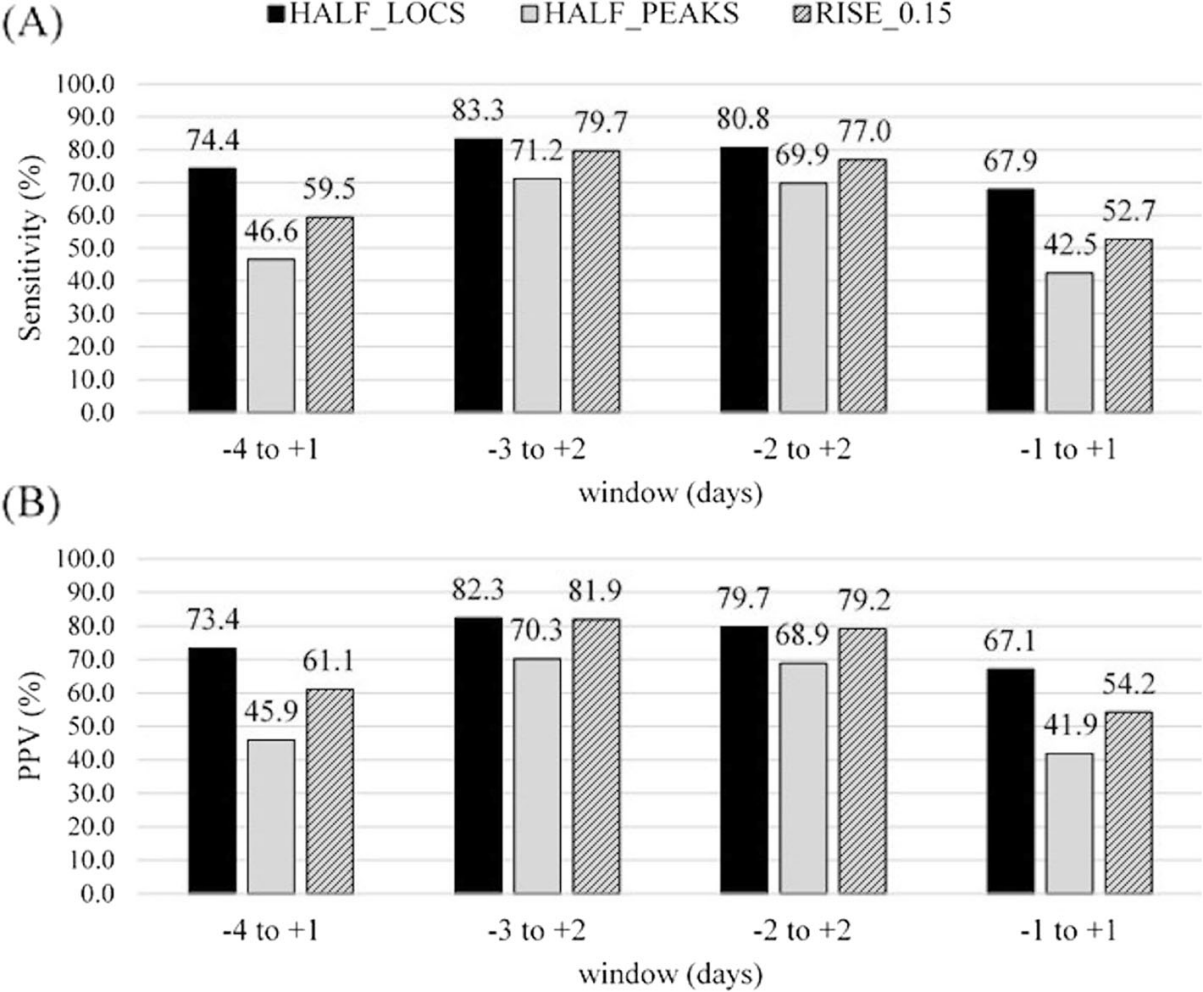
Ovulation prediction (**a**) sensitivities and (**b**) positive predictive values (PPV). Sensitivities and PPVs calculated for algorithms HALF_LOCS (TP + FN = 78), HALF_PEAKS (TP + FN = 73), and RISE_0.15 (TP + FN = 74) with different windows. The differences in the number of TP + FN are caused by the different data availability requirements of the algorithms

**Fig. 6:**
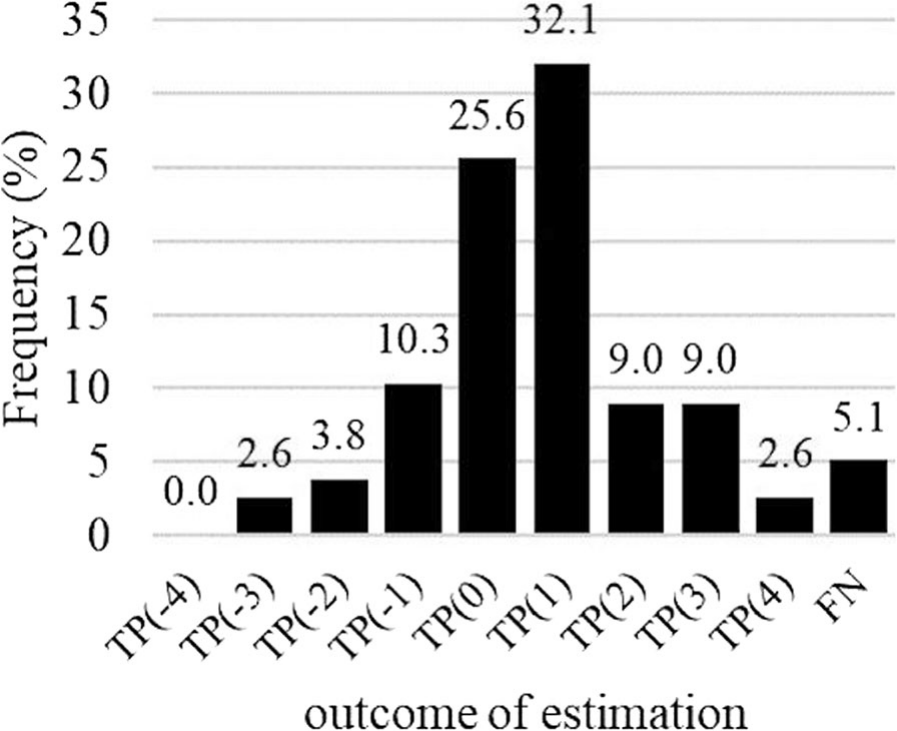
Ovulation prediction with algorithm HALF_LOCS. The distribution of detected ovulations (TP) in window ±4 days around the verified day relative to all reported ovulations (TP + FN = 78). FN represents ovulations not detected within the window

The number of ovulations analyzed for algorithms varied between 73 and 78. The algorithm HALF_LOCS had the highest and HALF_PEAKS the lowest sensitivity in all windows compared to other algorithms (Fig. 5a). Ovulation was detected with a mean offset of 0.6 days (SD 1.5) with the algorithm HALF_LOCS, 1.4 days (SD 1.5) with the algorithm HALF_PEAKS, and 0.6 days (SD 1.6) with the algorithm RISE_0.15 when the true positives in window of ±4 days around the verified ovulation day were used. With the best performing algorithm, HALF_LOCS, approximately 95% of ovulations were detected within ±4 days from the verified ovulation (Fig. 6), whereas the method based on biological rhythms detected only less than 80% of the ovulations within ±4 days (TP + FN = 80).

Two of the test subjects had BMI over 30, which is a potential confounder affecting distal skin temperature and the risk for menstrual disorders. One of them did not meet the pre-defined requirement of 0.15 °C difference between the phases (Fig. 3). After exclusion of these two subjects from algorithm testing the false negative percentage using window ±4 days decreased from 13.5% (Fig. 4) to 9.0% (TP + FN = 89) for menstruation detection and from 5.1% (Fig. 6) to 2.8% (TP + FN = 72) for the best performing algorithm, HALF_LOCS. When the 6-day fertility windows were concerned the sensitivity of HALF_LOCS increased from 74.4% (Fig. 5) to 79.2% for window − 4 to + 1 and from 83.3% (Fig. 5) to 86.1% for window − 3 to + 2.

## Discussion

In this study, the applicability of nocturnal finger skin temperature in monitoring menstrual cycle was evaluated, and algorithms for tracking the start of menstruation and ovulation were developed and tested in real life with a heterogeneous test group. Based on the results, the nocturnal finger skin temperature based on the Oura ring differed significantly between the follicular and luteal phases, with higher temperature in luteal phase. The nocturnal finger skin temperature correlated with oral temperature measured immediately after wake-up. The sensitivities and positive predictive values of algorithms for detecting menstruation and ovulation based on skin temperature were good with over 80% of the ovulations detected with the best-performing algorithm within a 6-day fertile window.

The findings support earlier ambulatory studies stating that different distal and proximal skin regions have a biphasic nature varying according to menstrual cycle phase [11, 22, 24]. Also, similar correlation between skin and morning oral temperatures has been found before in an ambulatory study using two-day mean temperatures of several skin regions and differences between phases with weighted mean temperatures of multiple skin regions. Similar to this study, skin temperatures had higher differences between phases than oral temperatures [11].

In this study, menstruation was detected with a sensitivity of 86.5 and 81.4% and a PPV of 85.6 and 81.4% for window lengths ±4 and ± 3 days, respectively. In an earlier study by Chen et al. [9] using abdominal skin temperature, a similar approach was used in algorithm evaluation, gaining a sensitivity of 91.8% and a PPV of 96.6% for menstruation detection using window length ± 3 days. However, the estimation offset was calculated relative to the closest day of menstrual flow whereas in this study, estimation offset was always calculated relative to the first day of menstrual flow.

The best-performing ovulation algorithm, HALF_LOCS, reached a sensitivity of 83.3% with fertile window from − 3 to + 2 days. This corresponds to earlier studies using wrist and in-ear wearables in temperature shift and fertile window detection and prediction [22–24]. However, it should be noted that at least in the studies of Shilaih et al. [22] and Luo et al. [23], the first day of the cycle was used whereas in this study, no background info on menstrual cycle day was used by the algorithms. Lou et al. [23] declared that some participants were uneager to wear the in-ear device for example during trips or periods or found it uncomfortable to sleep with the device.

In this study, the average offsets for the best-performing ovulation algorithm was 0.6 days from the day after the first positive LH test. These are in line with results from Berglund Scherwitzl et al. [30], who reported the mean delay of 1.9 days from the first positive ovulation test to the oral temperature-based estimation of the ovulation day.

The current ovulation tracking functionality might be a relevant additional feature in a ring-based health app that already provides feedback on sleep and physical activity around the clock. Compared to other widely used fertility tracking methods, such as applications requiring daily oral temperature recordings (Natural Cycles, Kindara, etc.), wearables offer an effortless new way of measuring the temperature continuously.

There were some limitations in the study. The number of participants was rather low, so a further study is needed to show the generalizability of the results. This pilot was designed to evaluate the applicability in real-life and thus the inclusion and exclusion criteria were quite loose, which resulted in quite a versatile group of women with wide range of age, obesity, different underlying diseases, continuous medications, and irregular cycles. However, when two obese test subjects were excluded from the algorithm testing, the results suggested an improved performance of the method.

For oral temperature measurements, the exact same measurement time for each day to minimize the effect of the circadian rhythm was not demanded, which could be one reason for the high standard deviation of the daily temperature values and which may decrease the reliability of the measurement as a reference for temperature in the correlation analyses. The standard deviation of daily skin temperatures was also high and even slightly higher than for oral, which could be explained by the effect of environmental factors, such as changes in ambient temperature between the nights [15]. There are also other factors influencing skin temperature values, such as smoking [31], but since we did not have any smokers in our study, these results cannot be generalized to smokers. It is also true that obese people have generally higher finger skin temperature, and obesity increases the risk for menstrual disorders. Additionally, some underlying diseases and medications may have effect on temperature or cycle.

The Oura ring temperature sensors were not calibrated before measurements, so the absolute daily values could not be utilized in preprocessing of the data. However, as one of this study’s strengths, the rings provided a really practical way to measure skin temperature for a longer period of time.

In algorithm design, the start of menstruation and ovulation day were tracked using data from the whole study period. In the future, it would be more useful and practical to develop algorithms to predict the dates based on the data measured preceding the date to be predicted, and utilizing machine learning algorithms and user input on menstruations in order to achieve personalized functionality.

## Conclusion

This pilot study suggests that nocturnal finger skin temperature based on the Oura ring has potential to be used in menstrual cycle phase monitoring in ambulatory conditions. However, further larger studies to validate the applicability are needed. The tested algorithms had good sensitivity and positive predictive values in menstrual cycle phase tracking. Positive study outcomes encourage further development of the menstrual cycle phase detecting algorithms, as their performance could be improved by utilizing machine learning algorithms and adding other physiological metrics to the estimation models.

In the future, technologies for monitoring menstrual cycle may be applicable, in addition to obvious use cases in fertility window tracking, for different kinds of personalized and persuasive systems developed to support changes in human behaviors, such as in sports routines, weight management, and smoking cessation.

## Abbreviations

AVG_MCL: Average menstrual cycle length
BBT: Basal body temperature
CBT: Core body temperature
FFT: Final fertile phase
FN: False negative
FP: False positive
IIP: Initial infertile phase
LH: Luteinizing hormone
MAX: Maximum
MF: The mean temperature of the follicular phase
MIN: Minimum
ML: The mean temperature of the luteal phase
NTC: Negative temperature coefficient
PPV: Positive predictive value
r: Correlation coefficient
rmcorr: Repeated measures correlation
SD: Standard deviation
TP: True positive
